# Low program access despite high burden of sexual, structural, and reproductive health vulnerabilities among young women who sell sex in Mombasa, Kenya

**DOI:** 10.1186/s12889-020-08872-6

**Published:** 2020-05-29

**Authors:** Elizabeth Roberts, Huiting Ma, Parinita Bhattacharjee, Helgar K. Musyoki, Peter Gichangi, Lisa Avery, Janet Musimbi, Jenkin Tsang, Shem Kaosa, Japheth Kioko, Marissa L. Becker, Sharmistha Mishra, on behalf of the Transitions Study Team

**Affiliations:** 1 MAP Centre for Urban Health Solutions, Li Ka Shing Knowledge Institute, St. Michael’s Hospital, University of Toronto, Toronto, Canada; 2 Centre for Global Public Health, University of Manitoba, Winnipeg, Canada; 3 Technical Support Unit, Partnership for Health and Development in Africa, Nairobi, Kenya; 4 National AIDS and STI Control Programme, Nairobi, Kenya; 5 International Centre for Reproductive Health-Kenya, Mombasa, Kenya; 6 Department of Medicine, Division of Infectious Disease, University of Toronto, Toronto, Canada; 7 Institute of Medical Sciences, University of Toronto, Toronto, Canada; 8 Institute of Health Policy, Management, and Evaluation, University of Toronto, Toronto, Canada

**Keywords:** Sex work, Kenya, Sustainable development goals, HIV, Sexual health, Reproductive health, Sexual violence, Physical violence, Adolescence, Youth

## Abstract

**Background:**

Across Sub-Saharan Africa, young women who sell sex (YSW) face institutional barriers in accessing sexual health and HIV prevention programs designed for female sex workers. In 2018, Kenya developed a national framework to guide service provision for YSW aged 14–24 years. To help inform the implementation of the framework, we estimated the burden of vulnerabilities related to the Sustainable Development Goals (SDGs related to health and gender equality) and program contact among YSW.

**Methods:**

We used data from Transitions, a 2015 bio-behavioural cross-sectional survey of 408 YSW aged 14–24 years in Mombasa, Kenya. We estimated the prevalence of sexual (inconsistent condom use), structural (financial, violence), and reproductive health vulnerabilities; and characterized engagement with local HIV programs tailored to sex workers. We then compared the prevalence of vulnerabilities by age group (14–18 years, *N* = 117; 19–24 years, *N* = 291) and by program contact (ever contacted by local program for sex workers).

**Results:**

47.3% reported inconsistent condom use with any partner in the previous week (no difference by age-group, *p* = 1.00). Structural vulnerabilities were common and did not vary by age: 83.6% did not have a regular source of income; 29.9 and 29.2% had experienced physical and sexual violence, respectively. 26.5% reported at least one pregnancy before age 18, and 18.5% used a non-reliable form of contraception with little variability by age. 25.7% were aware of at least one program, and only 13.7% of YSW had ever been contacted by a program (8.5% of those aged 14–18 years; and 15.8% of those aged 19–24 years, *p* = 0.06). Sexual, structural, and reproductive health vulnerabilities did not vary by program contact.

**Conclusions:**

SDG-related vulnerabilities begin early in the lives of YSW who are not currently reached by programs designed for female sex workers.

## Background

In Sub-Saharan Africa (SSA), adolescent girls and young women (AGYW) experience disproportionately high rates of HIV. Women aged 15–24 years bear 26% of the burden of new HIV infections among adults in SSA [1]. Meanwhile, in Eastern and Southern Africa, women who sell sex are three times more likely to be living with HIV than other women of reproductive age [2]. Although less is known about young women who sell sex (YSW), a growing body of literature from SSA suggests a high incidence and burden of HIV by age 24 among YSW stemming from early experience of sexual, structural, and reproductive health vulnerabilities [3–8].

In Kenya, the median age of AGYW self-identifying as a sex worker or entering sex work ranges from 14 to 18 years, which means the first years of formal sex work occur during adolescence [9, 10]. Many sexual, structural, and reproductive health vulnerabilities reported by YSW in Kenya start as early as first sex and are often associated with entry into sex work at an earlier age [11–15]. Emerging data also suggest that YSW experience higher rates of violence and substance use compared with older women who sell sex [15].

Most HIV prevention and sexual health programs tailored to sex workers are not designed to reach YSW. The United Nations Convention on the Rights of the Child classifies YSW under age 18 years as sexually exploited persons, thereby mandating that states report adolescents who sell sex [16, 17]. While this is an important step to reduce sexual exploitation of children and adolescents, the requirement to report could deter YSW from seeking preventative services – especially those who fear detention for other reasons [15]. Meanwhile, school-based sexual and reproductive health programs may overlook YSW who have terminated their studies and may not sufficiently address vulnerabilities associated with sex work [15]. Prior to 2018 in Kenya, guidance for sex worker programs restricted service provision to women over 17 years of age [18]. Program services include condom promotion, testing and either treatment or referral for treatment of HIV and other sexually transmitted infections, HIV pre-exposure prophylaxis, and violence support programs [19]. Services are delivered in the community by peer educators (former or current sex workers) through outreach as well as at facilities (drop-in centers or clinics) designed specifically for female sex workers [19]. Indeed, emerging data on YSW in Kenya led to development of its 2018 guidelines for service provision to key populations aged 14–24 years and included YSW as mature minors or emancipated minors [18]. Therefore, at the time of this study’s data collection in 2015, sexual, structural, and reproductive health services were not tailored to reach or serve YSW in Kenya.

The Sustainable Development Goals (SDGs) are a series of 17 goals set by the United Nations General Assembly in 2015 to be attained for 2030 which serves as a blueprint for improving global health [20]. Several SDG targets address sexual, structural and reproductive vulnerabilities. Kenya has prioritized these targets, including economic empowerment and reduced violence in accordance with SDG-5, which strives to achieve gender equity and empower all women and girls [21]. HIV remains a leading cause of death among women of reproductive age, and the *Kenya AIDS Strategic Framework* and *Kenya’s Fast Track Plan to End HIV and AIDS Among Adolescents and Young People* align with the goals of SDG-3, which aims to ensure health lives and promote well-being for all at all ages [22, 23]. Although YSW are not explicitly included as a vulnerable group within the population of AGYW, their vulnerabilities are intrinsically linked to the SDGs [15]. Indeed, there is a growing call to understand the unmet needs of YSW to guide gender-responsive approaches that are grounded in human-rights principles to meet the SDGs [18, 24].

As sex worker programs across Kenya develop strategies to implement the 2018 recommendations for YSW, we sought to identify the existing SDG-related vulnerabilities and program reach using secondary data from the *Transitions* study of YSW in Mombasa.

## Methods

Our aims were to: (1) estimate the prevalence of vulnerabilities related to sexual health (inconsistent condom use), structural factors (social economics, violence, and alcohol use), and reproductive health (pregnancy, abortion, and contraception) among YSW in Mombasa, Kenya in 2015; (2) estimate the level of engagement with programs designed for female sex workers in 2015; and (3) determine if vulnerabilities vary by age and by contact with programs designed for female sex workers.

### Study setting and population

We used data from a behavioural cross-sectional survey, administered between April and November 2015, to women aged 14–24 years who were congregating at sex work venues (hotspots) in Mombasa, Kenya. Sex work venues include any locale where individuals may solicit clients for sex in exchange for money, and could include nightclubs, hotels, bars, guest houses, lodges, restaurants, local brew dens, sex dens and brothels, or streets and other public spaces [25]. Eligibility criteria for the survey included: cis-gender females, aged 14–24 years, who had ever had vaginal or anal sex and were able to provide written informed consent. For the current study on YSW, we restricted our analyses to data on participants who answered yes to at least one of the following questions in the questionnaire: “*Presently, do you consider yourself a sex worker*?” or “*Were you ever a sex worker*?”

### Study design and data collection

We identified hotspots using geographic mapping to enumerate the distribution and population size of YSW, which led to the sampling frame and hotspot sampling via probability proportional to estimated YSW population size at each hotspot. Peer/community researchers (i.e. current or former sex workers engaged with local HIV prevention programs) invited potential participants for eligibility screening and consent as detailed previously [11]. Trained interviewers used structured questionnaires to conduct face-to-face interviews in English or Kiswahili. The questionnaire included information on sociodemographic characteristics, sexual behaviours, structural factors, reproductive behaviours and program engagement [11]. The questionnaire was developed for the purpose of this study, and pilot-tested with local community and programmatic input, and data were collected in partnership with the International Centre for Reproductive Health Kenya. Details of the data collection have been detailed previously [11, 25, 26].

### Measures - vulnerabilities

We selected SDG-relevant vulnerabilities in three domains: sexual health in relation to condom use; structural vulnerabilities; and reproductive health. Vulnerabilities were chosen in accordance with the SDGs and availability of variables collected in the survey. The questions from the survey used to generate each variable are included in Additional file 1.

Sexual vulnerabilities included: inconsistent condom use, defined as at least one encounter (vaginal and/or anal sex) without a condom, with any partner in the previous week. We then defined inconsistent condom use with a paying partner (at least one vaginal and/or anal sex encounter without a condom with a paying partner) and with a non-paying partner (at least one vaginal and/or anal sex encounter without a condom with a non-paying partner) among those who had sex with a paying and non-paying partner in the previous week, respectively.

Structural vulnerabilities were conceptualized as per Shannon et al. where factors operating beyond the individual-level influence health and wellbeing [27]. Structural vulnerabilities were divided into: socioeconomic; experience of sexual partner or police violence; and alcohol-related [2, 28, 29]. We defined socioeconomic vulnerabilities as those that limit financial independence: (a) inconsistent (irregular) source of income including sex work; (b) could not fully cover last month’s living expenses; (c) did not keep all wage from last month’s sex work (i.e. percentage of money from sex work is given to person who arranged the exchange); (d) illiteracy (i.e. cannot read and/or write); (e) has not completed primary school.

Violence-related structural vulnerabilities included: lifetime (“ever”) and recent (“past year”) experience of physical and sexual violence perpetrated by any sexual partners (clients and non-clients). Police harassment (recent and lifetime) was defined separately as physical abuse or arrest, perpetrated by law enforcement, including the police and *“sungu sungu”* (community policing groups) [30].

Alcohol- related structural vulnerabilities included: (a) alcohol consumption in past month; (b) inebriation in past month; (c) sex while inebriated in past month; (d) sex with an inebriated partner in past month. Inebriation was defined as reporting self or partner was “*drunk*” (under the influence of alcohol).

Reproductive health vulnerabilities included: (a) history of adolescent pregnancy, defined as first pregnancy before 18 years of age; (b) history of induced abortion; (c) the most recent induced abortion was unsafe; (d) never used contraception; (e) current use of unreliable contraception. Induced abortions were defined as unsafe if conducted in the absence of trained health workers and/or in the following venues: “[respondent’s] *home, someone else’s home, unlicensed clinic (e.g. quack)*” [31]. Safe induced abortions included those conducted in public, private and government health facilities [31]. We defined unreliable contraception as inconsistent and non-modern forms of contraception (i.e “*rhythm method*”, “*lactational amenorrhea”, “emergency contraception” and “withdrawal”*) [32].

### Definition - program engagement

Programs refer to local non-governmental organization, community-based organization and/or faith-based organization programs that provide services to female sex workers in Mombasa. We examined various levels of program engagement: (a) aware of a program; (b) ever contacted by a program; (c) currently registered in a program; (d) ever used a program clinic; (e) currently working (paid employment) or volunteering as a peer worker in a program. In the questionnaire, items surrounding program registration, clinic use, and status as a peer worker were restricted to participants who had ever been contacted by a program.

### Statistical analysis

We used descriptive statistics to report the prevalence and 95% confidence intervals (CIs) of vulnerabilities and of program engagement. We compared the difference in prevalence of each vulnerability and of program engagement by age group (14–18 years; 19–24 years). We also examined the prevalence of vulnerabilities by whether the participant has ever been contacted by programs. We used the χ^2^ and Fisher’s exact tests for the comparison of proportions as appropriate. We used R version 3.4.4 for statistical analyses and graphics.

## Results

### Participant characteristics (Table 1)

Of the 1419 women invited to participate, 1304 were eligible, and of whom 1299 provided consent and completed the interview (91.5% of those screened, 99.6% of those eligible). Four hundred and eight self-identified as sex workers, of whom 95.8% (*N* = 391) identified as current sex workers, and the remaining (*N* = 17) identified as past sex workers.

**Table 1:** Characteristics of women age 14–24 years who sell sex in Mombasa, Kenya (2015)

Characteristics	*N* = 408	Median (IQR)	% (95% CI)
**Current age (years)**			
Median age (IQR)	–	20 (18–22)	–
Age-group			
14–18	117	–	28.7 (24.3–33.1)
19–24	291	–	71.3 (66.9–75.7)
**Characteristics of sexual partnerships outside of sex work**			
Ever married	27	–	6.6 (4.2–9.0)
Had at least one non-paying sexual partner in past month	244	–	59.8 (55.0–64.6)
Median number of non-paying sexual partners in past month (IQR)^a^	–	2 (1–4)	–
**Characteristics of sex work**			
Age of first sex, years	–	15 (14–17)	–
Age of first paid sex^b^, years	–	18 (16–20)	–
Duration in sex work, years	–	2 (1–3)	–
Had at least one paying sexual clients in past month	328		80.4 (76.5–84.2)
Median number of paying sexual partners in past month (IQR)^c^	–	7 (4–15)	–
**Reproductive History**			
Ever pregnant	234	–	57.4 (52.6–62.2)
Currently has > = 1 child^d^	188	–	46.1 (41.2–50.9)

*CI* confidence interval, *IQR* inter-quartile range

^a^Among participants who had at least one non-paying sexual partner in the past month (*N* = 244)

^b^Paid sex is defined as the first time when a participant self-identified as a sex worker

^c^Among participants who had at least one paying sexual partners in the past month (*N* = 328)

^d^Answer was > = 1 to the question: “how many children have you given birth to who are alive, but may or may not live with you”

The median age of participants was 20 years (inter-quartile range [IQR] 18–22); and 28.7% (*N* = 117/408) were between the ages of 14–18 years (Table 1). Few (6.6%, *N* = 27/408) were ever married. More than half (59.8%, *N* = 244/408) reported having sex with a non-paying sexual partner in the past month, with a median of 2 (IQR 1–4) non-paying sexual partners in the past month. The median age of first sex was 15 years (IQR 14–17). The median age at which participants self-identified as a sex worker was 18 years (IQR 16–20), with a median duration in formal sex work of 2 years (IQR 1–3). Four out of five (80.4%, *N* = 328/408) participants had at least one paying sexual clients in the past month. The median number of paying partners in the previous month was 7 (IQR 4–15). Over half of the participants (57.4%, *N* = 234/408) had been pregnant at least once.

### Program engagement by age (Fig. 1)

**Fig. 1:**
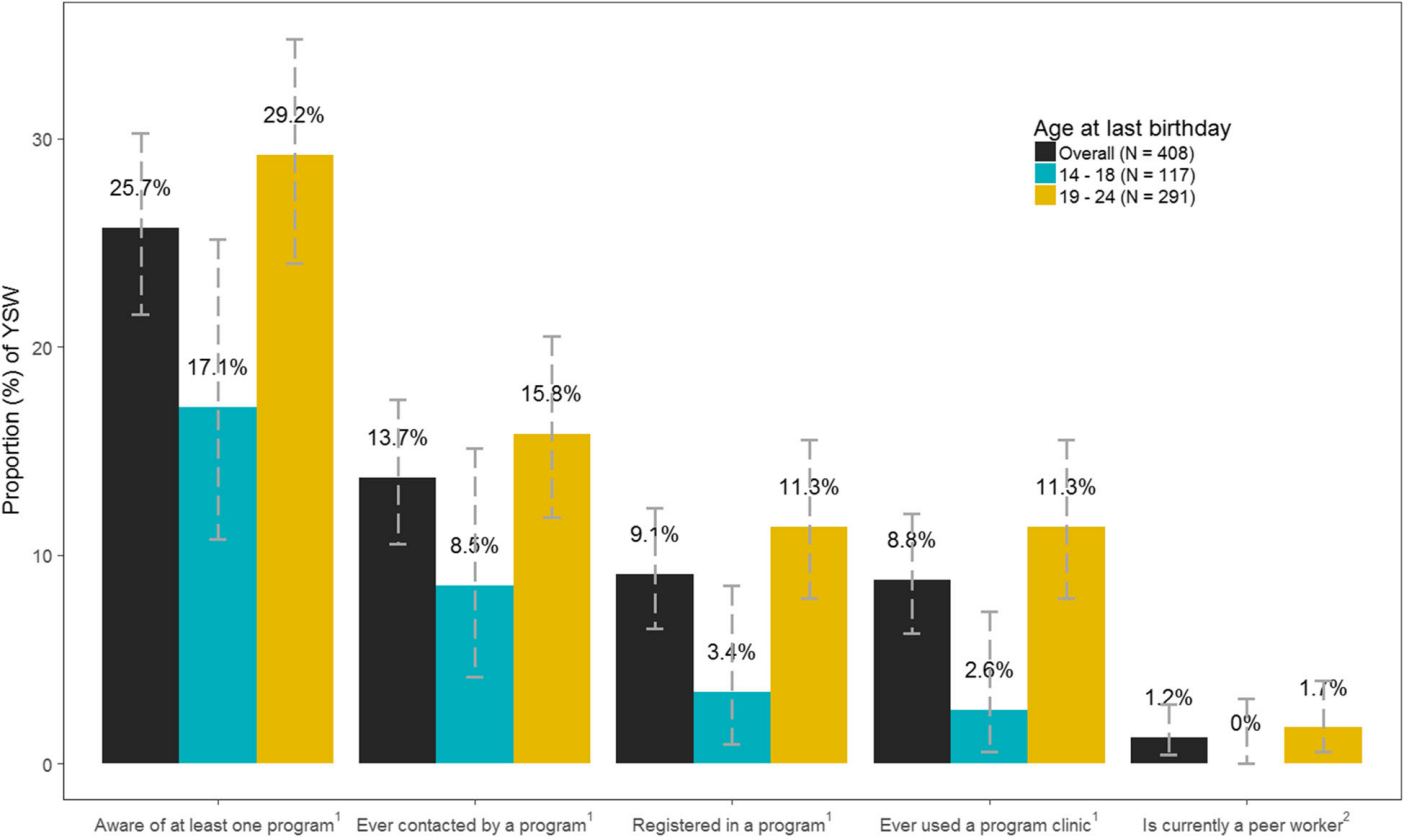
Program engagement among women age 14–24 years who sell sex in Mombasa, Kenya (2015). ^1^Program defined as a non-governmental organization /community-based organization. ^2^Peer worker of a non-governmental organization /community-based organization

Overall, program awareness and program contact were low (Fig. 1). One quarter of participants (25.7%, *N* = 105/408) were aware of at least one program. 13.7% (*N* = 56/408) were ever contacted by a program, 9.1% (*N* = 37/408) were registered in a program, and 8.8% had ever used a program clinic (*N* = 36/37; 97.3% of those who were registered). Only 1.2% (*N* = 5/37; 13.5% of those registered) were peer workers. In general, program awareness (29.2% vs. 17.1%, *p* = 0.01) and program contact (15.8% vs. 8.5%, *p* = 0.06) were higher among participants aged 19–24 years compared to those aged 14–18. 3.4% (*N* = 4/117) of participants aged 14–18 and 11.3% (*N* = 33/291) of participants aged 19–24 were registered with a local program. With respect to later elements of program engagement, among YSW, 2.6% of those aged 14–18 years and 11.3% of those aged 19–24 years had ever used a program clinic (*p* <  0.01).

### Sexual vulnerabilities by age (Table 2)

Nearly half of the participants who had sex with any partner in the previous week (47.3%, *N* = 160/338) reported inconsistent condom use. With respect to inconsistent condom use with at least one sexual encounter, 29.6% (*N* = 86/291) and 55.8% (*N* = 115/206) of participants reported this behaviour with any paying partner and with any non-paying partner respectively. The prevalence of inconsistent condom use was similar across the two age-groups, irrespective of partner type (Table 2).

**Table 2:** Inconsistent condom use in the past week among YSW by age group and program contact in Mombasa, Kenya (2015)

	Inconsistent condom use in previous week, by partner type^**a**^							
	Overall population		Age group			Program contact^**b**^		
Sexual engagement in previous week, by partner type			14–18, *N* = 117	19–24, *N* = 291		No, *N* = 352	Yes, *N* = 56	
	n/N	% (95% CI)	% (95% CI)	% (95% CI)	*p-value*	% (95% CI)	% (95% CI)	*p-value*
With paying partner (*N* = 291)	86/291	29.6 (24.3–34.8)	32.5 (22.2–42.8)	28.4 (22.3–34.5)	0.56	29.8 (24.1–35.5)	28.3 (15.2–41.3)	0.97
With non- paying partner (*N* = 206)	115/206	55.8 (49.0–62.6)	57.7 (44.3–71.1)	55.2 (47.3–63.0)	0.87	56.0 (48.6–63.4)	54.8 (37.3–72.4)	1.00
With any partner^c^ (*N* = 338)	160/338	47.3 (42.0–52.7)	47.2 (36.8–57.6)	47.4 (41.2–53.6)	1.00	47.7 (42.0–53.5)	45.1 (31.4–58.8)	0.84

*YSW* young women who sell sex, age 14–24 years

^a^Inconsistent condom use defined as at least one encounter (vaginal and/or anal sex) without a condom with any partner of a given type in the previous week

^b^ Program contact defined as ever contacted by peers of a non-governmental organization /community-based organization

^c^Defined as inconsistent condom use during vaginal and/or anal sex with > = 1 sexual partner in the previous week

### Socioeconomic structural vulnerabilities by age (Table 3)

Most (83.6%, *N* = 341/408) participants did not have a regular source of income; 82.4% (*N* = 336/408) reported they could not independently cover their living expenses last month; although only 13.5% (*N* = 55/408) reported they did not keep all their wages. Few participants (2.7%, *N* = 11/408) could not read and/or write and 30.4% (*N* = 124/408) had not completed primary school. There was little variability in socioeconomic vulnerabilities by age, although there was a trend towards lower educational attainment among younger participants (Table 3).

**Table 3:** Structural vulnerabilities by age and program contact among YSW in Mombasa, Kenya (2015)

Structural vulnerability	Overall population		Age group			Program contact^**a**^		
		*N* = 408	14–18, *N* = 117	19–24, *N* = 291		No, *N* = 352	Yes, *N* = 56	
	n/N	%(95% CI)	%(95% CI)	%(95% CI)	*p-value*	% (95% CI)	% (95% CI)	*p-value*
**Socioeconomic**								
Does not have regular source of income (including sex work)	341/408	83.6 (79.6–87.0)	85.5 (77.8–91.3)	82.8 (78.0–87.0)	0.56	83.2 (78.9–87.0)	85.7 (73.8–93.6)	0.85
Cannot fully cover living expenses from last month’s sex work	336/408	82.4 (78.3–85.9)	77.8 (69.2–84.9)	84.2 (79.5–88.2)	0.15	82.4 (78.0–86.2)	82.1 (69.6–91.1)	1.00
Does not keep all wages from last month sex work	55/408	13.5 (10.3–17.2)	12.0 (6.7–19.3)	14.1 (10.3–18.6)	0.63	13.1 (9.7–17.0)	16.1 (7.6–28.3)	0.53
Has not completed primary school	124/408	30.4 (26.0–35.1)	34.2 (25.7–43.5)	28.9 (23.7–34.4)	0.34	30.7 (25.9–35.8)	28.6 (17.3–42.2)	0.88
Cannot read and/or write	11/408	2.7 (1.4–4.8)	3.4 (0.9–8.5)	2.4 (1.0–4.9)	0.52	2.8 (1.4–5.2)	1.8 (0.0–9.6)	1.00
**Violence**								
Physical violence by sexual partner, ever	122/408	29.9 (25.5–34.6)	29.1 (21.0–38.2)	30.2 (25.0–35.9)	0.90	30.4 (25.6–35.5)	26.8 (15.8–40.3)	0.64
Physical violence by sexual partner, past 1 year^b^	79/122	64.8 (55.6–73.2)	76.5 (58.8–89.3)	60.2 (49.2–70.5)	0.14	66.4 (56.6–75.2)	53.3 (26.6–78.7)	0.39
Sexual violence, ever	119/408	29.2 (24.8–33.8)	24.8 (17.3–33.6)	30.9 (25.7–36.6)	0.23	30.4 (25.6–35.5)	21.4 (11.6–34.4)	0.21
Sexual violence, past 1 past year^c^	58/119	48.7 (39.5–58.1)	55.2 (35.7–73.6)	46.7 (36.1–57.5)	0.52	47.7 (37.9–57.5)	58.3 (27.7–84.8)	0.55
Police harrassment^d^, ever	183/408	44.9 (40.0–49.8)	29.1 (21–38.2)	51.2 (45.3–57.1)	< 0.01	43.2 (37.9–48.5)	55.4 (41.5–68.7)	0.11
Police harrassment^d,e^, past 1 year	139/183	76.0 (69.1–82.0)	85.3 (68.9–95.0)	73.8 (66.0–80.7)	0.19	75.7 (68–82.2)	77.4 (58.9–90.4)	1.00
**Alcohol**								
Consumed alcohol in the last month	324/408	79.4 (75.2–83.2)	76.9 (68.2–84.2)	80.4 (75.4–84.8)	0.42	78.4 (73.7–82.6)	85.7 (73.8–93.6)	0.28
Consumed alcohol almost everyday	121/408	29.7 (25.3–34.3)	32.5 (24.1–41.8)	28.5 (23.4–34.1)	0.47	29.5 (24.8–34.6)	30.4 (18.8–44.1)	0.88
Inebriated^f,g^ in the last month	154/324	47.5 (42.0–53.1)	46.7 (36.1–57.5)	47.9 (41.3–54.5)	0.90	45.7 (39.7–51.7)	58.3 (43.2–72.4)	0.12
Inebriated^f,h^ during sex in the last month	112/154	72.7 (65.0–79.6)	78.6 (63.2–89.7)	70.5 (61.2–78.8)	0.42	74.6 (66.1–81.9)	64.3 (44.1–81.4)	0.35
Partner inebriated^f^ during sex in the last month	283/408	69.4 (64.6–73.8)	65 (55.6–73.5)	71.1 (65.6–76.3)	0.24	69 (63.9–73.8)	71.4 (57.8–82.7)	0.76

*CI* confidence interval, *YSW* young women who sell sex, age 14–24 years

^a^Program contact defined as ever contacted by peers of a non-governmental organization/community-based organization

^b^Among participants who ever had experienced physical violence by sexual partner (*N* = 122)

^c^Among participants who ever had experienced sexual violence (*N* = 119)

^d^Police harassment was defined as experiencing physical assault or arrest by law enforcement while working as a sex worker

^e^Among participants who ever had experienced police harassement (*N* = 183)

^f^Inebriated defined as answering at least one time to the following question: “In the last month, how many times did you get drunk (under the influence of alcohol)?”

^g^Among participants who consumed alcohol in the last month (*N* = 324)

^h^Among participants who self-declared to be inebriated in the last month (*N* = 154)

### Violence-related structural vulnerabilities by age (Table 3)

Findings show that 29.9% (*N* = 122/408), 29.2% (*N* = 119/408), and 44.9% (*N* = 183/408) of participants reported ever experiencing physical violence, sexual violence, and police harassment respectively (Table 3). Of those, 64.8% (*N* = 79/408), 48.7% (*N* = 58/408), and 76.0% (*N* = 139/408) experienced physical violence, sexual violence, and police harassment, respectively in the previous year. The prevalence of physical (30.2% [*N* = 88/291] vs. 29.1% [*N* = 34/117], *p* = 0.90) or sexual violence (30.9% [*N* = 90/291] vs. 24.8% [*N* = 29/117], *p* = 0.23) did not vary by age and 29.1 and 24.8% of participants had experienced physical and sexual violence at least once by age 18. The prevalence of police harassment was higher among older participants (lifetime experience, 51.2% [*N* = 149/291] vs. 29.1% [*N* = 34/117], *p* <  0.01).

### Alcohol-related structural vulnerabilities by age (Table 3)

Most (79.4%, *N* = 324/408) participants consumed alcohol in the past month (Table 3). Of those who consumed alcohol in the past month, 47.5% (*N* = 154/408) were inebriated at least once in the past month; and 72.7% (*N* = 112/154) reported they were inebriated at least once during sex in the past month. Most (69.4%, *N* = 283/408) also reported that at least one partner was inebriated during sex in the past month. As with the other structural vulnerabilities, the prevalence of alcohol-related vulnerabilities was similar in the two age-groups (*p* > 0.05 for each of the five measures, Table 3).

### Reproductive health vulnerabilities by age (Table 4)

About one in four participants (26.5%, *N* = 108/408) reported a history of at least one pregnancy during their adolescence. There was a higher prevalence of adolescent pregnancy among younger participants (37.6% [*N* = 44/117] vs. 22.0% [*N* = 64/291], *p* = 0.002). Among the 234 participants with a history of pregnancy, 23.9% (*N* = 56) reported at least one induced abortion in their lifetime. There was little variability by age such that by the age of 18 years, nearly a quarter of participants with a history of pregnancy reported at least one induced abortion. Two-thirds (66.1%, *N* = 37/56) of the most recent abortions were unsafe; with similar proportions across the age groups. Among those who were using contraception, 18.5% (*N* = 62/336) used a non-reliable form; with little variability by age group.

**Table 4:** Vulnerabilities in reproductive health by age and program contact among YSW in Mombasa, Kenya (2015)

	Overall population		Age group			Program contact^**a**^		
			14–18, *N* = 117	19–24, *N* = 291		No, *N* = 352	Yes, *N* = 56	
	n/N	% (95% CI)	% (95% CI)	% (95% CI)	*p-value*	% (95% CI)	% (95% CI)	*p-value*
Adolescent pregnancy^b^	108/408	26.5 (22.3–31.0)	37.6 (28.8–47.0)	22.0 (17.4–27.2)	< 0.01	25.3 (20.8–30.2)	33.9 (21.8–47.8)	0.19
Ever had abortion^c^	56/234	23.9 (18.6–29.9)	23.9 (12.6–38.8)	23.9 (18–30.7)	1.00	23.5 (17.8–30.0)	26.5 (12.9–44.4)	0.67
Most recent abortion was unsafe ^d, e^	37/56	66.1 (52.2–78.2)	81.8 (48.2–97.7)	62.2 (46.5–76.2)	0.30	66.0 (50.7–79.1)	66.7 (29.9–92.5)	1.00
Currently using unreliable forms of contraception^f, g^	62/336	18.5 (14.4–23.0)	18.1 (10.9–27.4)	18.6 (13.9–24.1)	1.00	17.4 (13.1–22.3)	24.1 (13.5–37.6)	0.25

*CI* confidence interval, *YSW* young women who sell sex, age 14–24 years

^a^ Program contact defined as ever contacted by peers of a non-governmental organization /community-based organization

^b^Adolescent pregnancy refers to the first pregnancy that occurred before age of 18

^c^Among participants who had a history of pregnancy (*N* = 234)

^d^Among participants who had a history of abortion (*N* = 56)

^e^Unsafe abortion defined as any abortion not performed in public/government/private/non-governmental organization /community-based organization/ faith-based organization facility

^f^Among participants who are currently using any forms of contraception (*N* = 336)

^g^Unreliable forms of contraception are defined as inconsistent and non-modern forms of contraception such as rhythm method, withdrawal, and emergency contraception

### Vulnerabilities by program contact (Tables 2, 3 and 4)

In general, there was little variability in the prevalence of sexual, structural and reproductive vulnerabilities by whether or not participants were contacted by local programs (Tables 2, 3, 4; *p* > 0.05 across each measure). For example, the prevalence of inconsistent condom use with any partner in the previous week was almost same among participants with and without program contact (28.3% vs 29.8%, *p* = 0.97). There were however a few notable differences with a suggestion of a trend towards higher or lower prevalence of structural and reproductive health vulnerabilities by program contact. For example, participants without program contact were more likely to report a lifetime history of sexual violence (30.4% vs 21.4%, *p* = 0.21) but those with program contact were more likely to report a lifetime history of police harassment (55.4% vs 43.2%, *p* = 0.11). Within the reproductive health domain, there was a trend towards higher prevalence of adolescent pregnancy (33.9% vs 25.3%, *p* = 0.19) among the small subset of participants who had been contacted by programs versus those who had not.

### Discussion

We found that YSW in Mombasa, Kenya experience a high burden of sexual (inconsistent condom use), structural (social economics, violence, and alcohol use), and reproductive vulnerabilities (pregnancy, abortion, and contraception), alongside low access and engagement in programs tailored for female sex workers. Although the prevalence of a few vulnerabilities, such as police harassment, was more commonly reported by YSW over the age of 18 years, the prevalence of most vulnerabilities was similar across age-groups. YSW under the age of 19 years were nearly half as likely to report program contact compared with YSW aged 19–24; and sexual, structural, and reproductive health vulnerabilities did not vary by program contact.

We examined vulnerabilities relevant to SDG-3 (health and wellbeing) and SDG-5 (gender equity and empowerment), and found that the prevalence of structural and reproductive vulnerabilities was higher than rates reported among AGYW of a similar age in Kenya. Eight in ten women aged 15–19 years surveyed in the household-based 2014 Demographic Health Survey reported they lacked employment - similar to the proportion of YSW 14–18 years of age in our study who reported irregular income [33]. However, the prevalence of irregular income was nearly twice as high among YSW aged 19–24 years in our study compared with the 47% of household survey participants aged 20–24 years who reported they lacked any income [33]. The prevalence of lifetime and of recent sexual violence among YSW in our study was three-fold higher than among 15–24 year old women in the household survey [33]. Three percent of AGYW consumed alcohol in the previous month, compared with nearly 8 in 10 YSW from our study [33]. Reproductive vulnerabilities, such as current use of unreliable contraceptives and adolescent pregnancy was nine-fold and three-fold higher among YSW than reported by 15–24 year old women in the household survey, respectively [33]. Induced abortions were also more common among YSW in our study compared with the national estimates of 38 and 76 per 1000 women aged 15–19 and 20–24 years respectively [34]. Taken together, the high prevalence across nearly all SDG-relevant vulnerabilities support earlier findings on heterogeneity in vulnerabilities across AGYW – and thus, this calls for prioritization and tailoring of services to meet the unique needs of this particularly vulnerable subset of AGYW [15].

The high and similar prevalence of lifetime experience of vulnerabilities among YSW across age groups suggests that SDG-relevant vulnerabilities begin early in the sexual life course of YSW and before the age of 18. Cohort effects are variations in characteristic(s) among a group of individuals over time and these individuals are grouped into a cohort based on their shared experience. Within the context of our study, this may have been the case with the trend observed with adolescent pregnancy – the prevalence of which was slightly higher among the ‘younger’ cohort of YSW born between 1997 and 2001 compared to those born between 1991 and 1996. This suggests that rates of adolescent pregnancy may be increasing over time among YSW. Cohort effects could also be a plausible reason for the higher lifetime prevalence of police harassment among older YSW given the 2014 scale-up of police education to reduce police-mediated violence and harassment against sex workers in Kenya [35]. Another reason for the difference in police harassment by age may be the potential for increased visibility of older sex workers to law enforcement. Finally, the high and similar prevalence of recent physical or sexual violence, and of police harassment among those who ever experienced it, across age-groups may be due to ongoing or repeated experience of these vulnerabilities over the sexual life-course of YSW – as previously shown with female sex workers in general across SSA [11, 36].

The potential reasons for the high burden of vulnerabilities among YSW, especially among those aged 14–18 years, are complex and inter-related. Age- and gender-based power dynamics with sex partners may be amplified in the context of sex work [14]. There may be fewer social connections with peers in the first few years of sex work leading to less social cohesion – and social cohesion has been associated with higher condom use [37]. Inebriation has been associated with physical and sexual violence perpetrated against AGYW [38] and among female sex workers [39]; and especially in the context of an inebriated partner [39]. Adolescent pregnancy is one of the leading causes of adolescent females dropping out of secondary school in Kenya [40], which in turn may limit their potential to become financially independent and also excludes women from school-based sexual and reproductive health education and services [16].

Nearly three in four YSW has yet to access sexual, structural, and reproductive health programs tailored to sex workers. Factors which could limit program engagement among YSW include barriers to self-disclosure to sex work programs and barriers faced by programs in reaching and providing services to YSW. YSW may also experience compounded stigma of being female sex workers as well as sexually-active adolescents or youth – potentially obstructing access not only to sex work programs [14], but also to sexual and reproductive health programs for women in general [41]. The 2018 guidelines for HIV programming for young key populations provides an important step in addressing the unmet needs identified in this study [18]. The guidelines deploy a rights-based reframing of services to empower programs to provide services once they reach YSW [18]. Our findings further contribute to the guidelines by providing estimates of the unmet needs with respect to SDG-related vulnerabilities, and signal that a venue-based approach may be helpful in identifying and reaching YSW. Indeed, the venue-based approach used in this survey suggests that YSW who are not currently accessing programs can be reached. And such ‘place-based’ approaches are now being trialed in other settings to reach YSW [42, 43]. The challenge thereafter lies in designing and evaluating the components of an effective program to address the unmet sexual, structural, and reproductive health needs of YSW [44]. YSW programming, alongside services for other young key populations, is gaining momentum in Kenya and the next few years will provide evidence to the effectiveness of reaching YSW and reducing vulnerabilities experienced by YSW [18]. Of note, our analysis did not examine use of services external to sex work programs. External services, like traditional clinic-based HIV testing services, would be considered in the design of programs for YSW so that the latter provide synergy and address the residual high prevalence of ongoing vulnerabilities identified in our study.

Study limitations include the use of self-reported data collected via face-to-face-interviews which are subject to recall and social desirability biases, respectively. Recall bias on dates in particular could lead to misclassification of recent experience of vulnerabilities. Social desirability bias could lead to under-reporting of vulnerabilities and potentially over-reporting of program engagement such that program access, while low, could still be an overestimate. We defined YSW based on self-identification as a sex worker; thus we excluded participants who have sex with men in exchange for money or gifts but who do not self-identify as sex workers. Our definition therefore, may lead to underestimating vulnerabilities and program contact. We were limited in our analyses to examining SDG-relevant vulnerabilities based on available data, and thus our findings do not represent the full spectrum of unmet needs among YSW that fall under the SDGs. Moreover, we only considered contact with programs tailored for women engaged in sex work, and not the wider set of services that women may use, such as the public sector family planning services. Important future work includes examining a wider spectrum of SDGs among YSW, access and use of services beyond those tailored for women engaged in sex work, and programmatic and implementation research into how best to reach YSW and address their vulnerabilities.

## Conclusion

In Kenya, there is a high burden of SDG-relevant vulnerabilities among AGYW who sell sex, starting well before the age of 18 years, and current sex work programs are not reaching them. To achieve the SDGs, a pragmatic and rights-based paradigm shift in sexual, structural, and reproductive health services is needed to reduce vulnerabilities at the intersection of adolescence and sex work.

## Supplementary information

**Additional file 1.** Survey questions from the *Transitions* Study used for generating variables. A list of survey questions from the *Transitions* Study that was used for generating variables.

## Data Availability

The individual-level data used and/or analysed for the current study, and the R script for analyses and figures, are available from the corresponding author on request. The external researchers or requesting parties will be asked to sign an *Agreement on Confidentiality and Use of Data* and will have virtual private network access to the anonymized, individual-level data which are securely stored at the Centre for Global Public Health Data Server at the University of Manitoba.

## Abbreviations

AGYW: Adolescent girls and young women
CI: Confidence interval
IQR: Inter-quartile range
SDG: Sustainable Development Goal
SSA: Sub-Saharan Africa
YSW: Young women who sell sex
