# Supplementary material for: Low program access despite high burden of sexual, structural, and reproductive health vulnerabilities among young women who sell sex in Mombasa, Kenya
Source: BMC Public Health. 2020 May 29;20:806. doi: 10.1186/s12889-020-08872-6 (PMC7257181; doi:10.1186/s12889-020-08872-6)
Supplement: Supplementary file 1 — Additional file 1. Survey questions from the Transitions Study used for generating variables. A list of survey questions from the Transitions Study that was used for generating variables. [file 12889_2020_8872_MOESM1_ESM.docx]

| **Variable** | **Survey Question** |
| --- | --- |
| **Sexual Variables** |  |
| Inconsistent condom use in the last week with paying partner(s) (among those who had paying partner(s) in the last week) | - How many PAYING CLIENTS did you have sex (vaginal, anal or both) with in the LAST WEEK OF SEX WORK? - Of the _____ PAYING CLIENTS with whom you had sex in the LAST WEEK OF SEX WORK, how many were REGULAR CLIENTS? - Of those REGULAR PAYING CLIENTS in the LAST WEEK OF SEX WORK, with how many of these men did you have: a) both vaginal and anal sex? And with how many of them was a condom NOT used? b) only vaginal sex? And with how many of them was a condom NOT used? c) only anal sex? And with how many of them was a condom NOT used? - Of those FIRST-TIME PAYING CLIENTS in the LAST WEEK OF SEX WORK, with how many of these men did you have: a) both vaginal and anal sex? And with how many of them was a condom NOT used? b) only vaginal sex? And with how many of them was a condom NOT used? c) only anal sex? And with how many of them was a condom NOT used? |
| Inconsistent condom use in the last week with non-paying partner(s) (among those who had non-paying partner(s) in the last week) | - Now I am going to ask you about ALL THE OTHER MEN with whom you had sex. These men may include NON-CLIENT SEX PARTNERS (i.e. men with whom you have sex, with the expectation that you would receive money, gifts or other resources in return but the price of sex is often not negotiated upfront and is implicitly understood) as well as HUSBAND, SPOUSE or BOYFRIENDS. How many OTHER MEN did you have sex with in the LAST WEEK? - Of the _____ OTHER SEX PARTNERS in the LAST WEEK, how many non-client sex partners did you have sex with who gave you money, gifts or other resources in exchange for sex? - Of those_____ non-client sex partners in the LAST WEEK that gave you money, gifts or other resources in exchange for sex, how many are REGULAR SEX PARTNERS with whom you had sex on more than one occasion? - Of those REGULAR, NON-CLIENT SEX PARTNERS in the LAST WEEK, with how many of these men did you have: a) both vaginal and anal sex? And with how many of them was a condom NOT used? b) only vaginal sex? And with how many of them was a condom NOT used? c) only anal sex? And with how many of them was a condom NOT used? - Of those FIRST-TIME NON-CLIENT SEX PARTNERS in the LAST WEEK, with how many of these men did you have: a) both vaginal and anal sex? And with how many of them was a condom NOT used? b) only vaginal sex? And with how many of them was a condom NOT used? c) only anal sex? And with how many of them was a condom NOT used? - Now let’s think about your husband, spouse or boyfriends. How many of these men did you have sex with in the LAST WEEK? - In the LAST WEEK, of your husband, spouse or boyfriends, with how many of these men did you have: a) both vaginal and anal sex? And with how many of them was a condom NOT used? b) only vaginal sex? And with how many of them was a condom NOT used? c) only anal sex? And with how many of them was a condom NOT used? |
| Inconsistent condom use in the last week with any partner (among those who had any partner in the last week) | - Inconsistent condom use in the last week with paying partner(s) (among those who had paying partner(s) in the last week) - Inconsistent condom use in the last week with non-paying partner(s) (among those who had non-paying partner(s) in the last week) |
| **Structural Variables** | |
| **Socioeconomic** | |
| Does not have regular source of income (including sex work) | Do you currently have a regular source of income? |
| Cannot fully cover living expenses from last month sex work | Did the money from your paying clients cover all living expenses last month? |
| Did not keep all wages from last month’s sex work | Some women only get to keep some of the money from their paying clients in exchange for sex and the rest of the money goes to the person who arranged the exchange. On average, in the LAST MONTH OF SEX WORK, how much of the money did you keep? |
| Has not completed primary school | What is the highest level that you completed in school? |
| Cannot read and/or write | Can you read or write? |
| **Violence** |  |
| Physical violence by sexual partner, ever | - Think of the FIRST TIME when you were physically hurt by a sex partner. How many years ago was that? If 2 years ago or less, in what month and year were you first physically hurt by a sex partner? - When was the LAST TIME a sex partner physically hurt you? |
| Physical violence by sexual partner, past 1 year |  |
| Sexual violence, ever | - Think of that FIRST TIME when the man forced you to have sex with him when you were not willing. Was that man your first sex partner? - How many years ago was it when this man forced you to have sex with him when you were not willing? If 2 years ago or less, in what month and year did this man force you to have sex with him when you were not willing? - When was the LAST TIME a man forced you to have sex with him when you were not willing? |
| Sexual violence, past 1 year |  |
| Police harassment, ever | - Think of the FIRST TIME when you were physically assaulted or arrested by law enforcement (e.g. police, sungu sungu etc.) while you were working as a sex worker. How old were you? - Think of the LAST TIME when you were physically assaulted or arrested by law enforcement while you were working as a sex worker. How old were you? - How old were you on your last birthday? |
| Police harassment, past 1 year |  |
| **Alcohol** |  |
| Consumed alcohol in the last month | Last month, how often did you consume alcohol? |
| Consumed alcohol almost every day in the last month | Last month, how often did you consume alcohol? |
| Inebriated in the last month | In the past month, how many times have you been drunk? |
| Inebriated during sex in the last month | In the past month, how often were you drunk when you had sex with: Regular/First-time clients/Regular/First-time non-clients, husband, spouse or boyfriend? |
| Partner inebriated during sex in the last month | In the past month, how often was this type of partner drunk when you had sex with him: Regular/First-time clients/Regular/First-time non-clients, husband, spouse or boyfriend? |
| **Reproductive Variables** |  |
| Adolescent pregnancy | - Have you ever been pregnant? - How old were you when you found out you were pregnant (FIRST PREGNANCY)? |
| Ever had abortion | How many abortions have you had? |
| Most recent abortion was unsafe | Where was the LAST ABORTION performed? |
| Currently using unreliable forms of contraception | There are various ways or methods that can be used to delay or avoid a pregnancy. What method(s) of contraception do you currently use (i.e. the last one month)? |
